# Comparison of survival between radiation therapy and trans-oral laser microsurgery for early glottic cancer patients; a retrospective cohort study

**DOI:** 10.1186/s40463-016-0155-1

**Published:** 2016-08-02

**Authors:** R. J. De Santis, I. Poon, J. Lee, I. Karam, D. J. Enepekides, K. M. Higgins

**Affiliations:** 1 Sunnybrook Health Sciences Centre, 2075 Bayview Avenue, Suite M1 102, Toronto, ON M4N 3 M5 Canada; 2 Department of Radiation Oncology, Sunnybrook Health Sciences Centre, University of Toronto, 2075 Bayview Avenue, Toronto, ON M4N 3 M5 Canada; 3 Department of Otolaryngology – Head & Neck Surgery, Sunnybrook Health Sciences Centre, University of Toronto, 2075 Bayview Avenue, Suite M1 102, Toronto, ON M4N 3 M5 Canada

**Keywords:** Trans-oral laser microsurgery, Radiation therapy, 5-year survival, Early glottic cancer, T1, T2

## Abstract

**Background:**

The literature reports various treatment methodologies, such as trans-oral laser microsurgery, radiation therapy, total/partial laryngectomies, and concurrent radiation chemotherapy for patients with early larynx cancer. However, at the forefront of early glottis treatment is trans-oral laser microsurgery and radiation therapy, likely due to better functional and survival outcomes. Here we conduct the largest Canadian head-to-head comparison of consecutive patients treated with either radiation therapy or trans-oral laser microsurgery. Additionally, we compare these two treatments and their 5-year survival rates post treatment to add to the existing literature.

**Methods:**

Charts of patients who were diagnosed with early glottic cancer between 2006 and 2013 were reviewed. Seventy-five patients were identified, and split into 2 groups based on their primary treatment, trans-oral laser microsurgery and radiation therapy. Kaplan–Meier survival curves, life-tables, and the log-rank statistic were reported to determine if there was a difference between the two treatment groups and their disease-specific survival, disease-free survival, and total laryngectomy-free survival. Additionally, each different survival analysis was stratified by potential confounding variables, to help conclude which treatment is more efficacious in this population.

**Results:**

The 5-year disease-specific survival rate is 93.3 % σ = 0.063 and 90.8 % σ = 0.056 for patients treated with trans-oral laser microsurgery and radiation therapy, respectively (*χ*^2^ < 0.001, *p* = 0.983). The disease free survival rate is 60.0 % (σ =0.121) for patients treated with trans-oral laser microsurgery, and 67.2 % (σ = 0.074) for those who received RT (*χ*^2^ = 0.19, *p* = 0.663). Additionally, the total laryngectomy-free survival rate is 84.1 % (σ = 0.1) and 79.1 % (σ = 0.072) for patients’ early glottic cancer treated by trans-oral laser microsurgery and radiation therapy, respectively (*χ*^2^ = 0.235, *p* = 0.628). Chi-square analysis of age-group versus treatment group (*χ*^2^ = 6.455, *p* = 0.04) and T-stage versus treatment group (*χ*^2^ = 11.3, *p* = 0.001) revealed a statistically significant relationship, suggesting survival analysis should be stratified by these variables. However, after stratification, there was no statistically significant difference between the trans-oral laser microsurgery and radiation therapy groups in any of the survival analyses.

**Conclusion:**

No difference was demonstrated in the 5-year disease-specific survival, disease-free survival, and total laryngectomy-free survival, between the RT and TLM treatment groups. Additionally, both groups showed similar 5-year survival after stratifying by confounding variables.

## Background

Deaths due to laryngeal cancer have been decreasing at a rate greater than 4 % each year since 2001 as reported by the Canadian Cancer Society. However, this figure, as explained by the Canadian Cancer Society, is largely due to the reductions in tobacco use rather than improvements in treatment [1]. Though this statistic is encouraging, more research is needed to evaluate best practice for larynx cancers to further help improve the survival rate of patients afflicted by these cancers. Standard care for glottic cancers staged T1 or T2, or early glottic cancer, is still up for contention. The literature reports various treatment methodologies, such as trans-oral laser microsurgery (TLM), radiation therapy (RT), total/partial laryngectomies, and concurrent radiation chemotherapy. However, at the forefront of early glottis cancer treatment is TLM and RT, likely due to better functional and survival outcomes. This study seeks to compare TLM and RT in early glottic cancer patients treated at Sunnybrook Health Sciences Centre., by analyzing their 5-year disease-specific survival, disease-free survival, and total laryngectomy-free survival.

Though several studies have compared TLM and RT, there seems to be a lack of consideration of potential confounding variables, and how they may affect the results. This report will identify 3 potential variables that may confound the results, test for their significance in the dataset, and stratify the analysis based on them if it proves reasonable. This will help to ensure that the results are showing what they are intended to, and avoid false conclusions. Mainly this study seeks to add more current data to the body of literature as the largest Canadian head-to-head comparison of consecutive patients treated with either RT or TLM, in order help make conclusions about which treatment should be used.

## Methods

### Patients

Charts of consecutive patients who were diagnosed with early glottic cancer between 2006 and 2013 were reviewed. Charts were collated from a list created by Sunnybrook Heath Science Centre otolaryngology research team by searching for all glottic cancer patients who were staged as T1 or T2. Cancer staging was done by pathologists at Sunnybrook Health Sciences Centre. Patients were only included in the study if their primary treatment was TLM or RT. All patients were given the option of either RT and TLM, and were treated under the care of a multidisciplinary team for head and neck cancer at Sunnybrook Health Sciences. Eighty-four patients were identified, and evaluated by a single reviewer. Four of the patients were excluded since their primary treatment did not include exclusively RT or TLM, 3 were excluded since the primary treatment was not available in the charts, and 2 were excluded since their T-stage was not available. This project was approved by the Research Ethics Board of Sunnybrook Health Science Centre (206–2009).

### Data collection

All data was collected by a single reviewer from the collated patient charts. Data extracted from the charts included demographic information, the patients’ initial treatment, T-stage, length of follow-up, and time until the patient died of the disease. Time measurements were calculated starting from the completion of primary treatment until death, or last follow-up date. Minimum follow-up was 1-month.

### Interventions

All patients in the radiation group were treated with intensity-modulated radiation therapy (IMRT). All patients in the surgery group were under the care of the attending surgeons KH or DE, and were treated with a CO2 laser.

### Statistical analysis

Statistical analysis was undertaken using SPSS® (V20, IBM Corp ©). Statistical significance for all tests were set at *p* < 0.05. Survival analyses were carried out using the Kaplan–Meier method, and the curves were compared using the log-rank statistic. Survival rates were obtained by calculating the cumulative proportion of patients alive at 5 years, accounting for censored data. Additionally, Life-tables were produced to aid in the interpretation of the survival curves. Afterwards, age, T-stage, and gender were tested to see if they were confounding variables using the Chi-square statistic. When comparing age to treatment modality, age was split into 3 groups, below 65, 65–75, and above 75. Age was split into three groups to allow for the comparison of the two treatments on the middle-aged (below 65), the “young-old” (65–75), and the “older-old” (above 75). The age cut-offs were set to create approximately equal amounts of patients in each age group. Depending on the results of the tests for confounding variables, survival analyses were stratified by the variables proving to be confounders and subsequently calculated as explained above. The primary outcomes are disease-specific survival, disease-free survival, and total laryngectomy-free survival. Survival time is measured from the completion of treatment until an event or the end of the 5-year follow-up is reached. The definition of the event for each of the 3 survival analyses is reported in the subsection below: Survival Analyses. Patients’ who did not experience an event but were lost to follow-up before the 5-year mark were used in the survival analysis until they were lost to follow-up, subsequently they were censored.

Demographic data that were continuous variables (smoking pack-years and age), were analyzed by a *T*-test to determine their mean in each treatment group and how they were distributed between TLM and RT groups. Smoking status, being a binary variable, was tested for equal distribution between the groups using chi-square analysis. Additionally, the mean radiation dosage for the RT group was reported.

### Survival analyses

Three types of survival analyses were conducted to compare the effectiveness of TLM and RT; disease-specific survival, disease-free survival, and total laryngectomy-free survival. Disease-specific survival analysis considered an event as death from the primary cancer. Disease-free survival analysis was conducted by classifying an event as the recurrence of the primary cancer or death from the primary cancer. Moreover, total laryngectomy-free survival was conducted under the assumption that an event occurred if a patient required a total laryngectomy or died from their primary cancer.

## Results

**Fig. 1:**
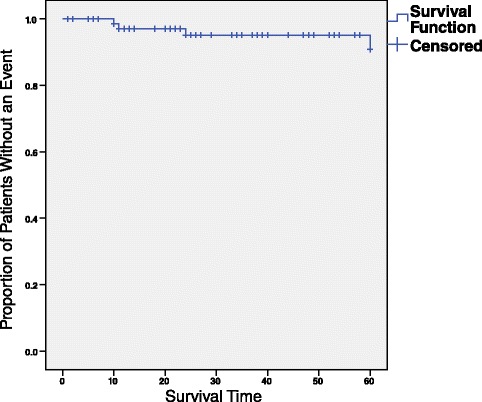
Overall 5-year survival curve. This survival curve depicts the 5-year survival for all patients reported in this study. Censored patients are those whose did not die from the cancer but were lost to follow-up before the 5-year mark

In total 75 patients, 31 % (23/75) treated with TLM and 69 % (52/75) by RT, were examined. Patient characteristics are listed in Tables 1 and 2. The overall 5-year survival rate, regardless of treatment is 90.8 % σ = 0.05 (Fig. 1). To proceed to survival analysis comparing the two treatment groups, it is first necessary to determine whether potential confounding variables are balanced between the two groups. Thus, 3 variables were tested to see if a statistically significant relationship existed between them and the treatment groups, the independent variable. If one does exist, the uneven distribution of patients based on the potential confounder could account for the observed results for 5-year survival of patients with early larynx cancer. However, before stratifying, there needs to be a reasonable explanation of why that variable can affect the outcome. Both of these criteria must be met in order to stratify by a variable in the survival analysis.

**Table 1:** Overall Subject Characteristic

Number of Patients	75
Age Range	42–91, mean = 68
Gender M:F	22:3
T1	47
T2	28
Age Group Distribution	<65 : 25
	65–75 : 28
	>75 : 22
Radiation Dosage for RT group	46 Gy-70 Gy
	mean = 54.71 Gy

**Table 2:** Subject Characteristics Stratified by Treatment Group

	TLM	RT	Statistical Significance
Age	mean = 72.5	mean = 66.5	*p* = 0.03
Age Group:	<65 : 4	<65 : 21	*p* = 0.04
	65–75 : 8	65–75 : 20	
	>75 : 11	>75 : 11	
T Stage	T1 = 21	T1 = 26	*p* = 0.001
	T2 = 2	T2 = 26	
Smoking Status	Non-smoker: 7	Non-smoker: 5	*p* = 0.054
	Smoker: 16	Smoker: 47	
Smoking Pack-Years	mean = 20.2	mean = 31.7	*p* = 0.069
Gender	Male: 20	Male: 46	*p* = 1.00-
	Female: 3	Female: 6

**Fig. 2:**
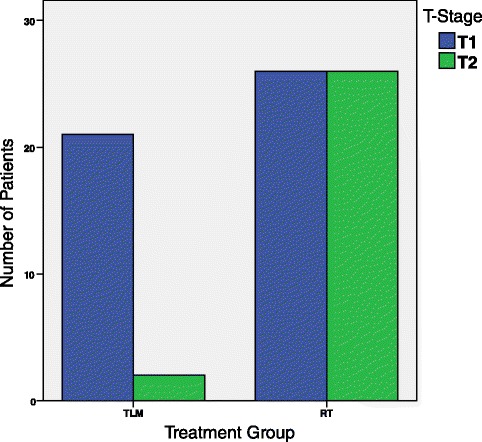
Distribution of patients by treatment group stratified by T-Stage. This double bar graph represents the distribution of patients who underwent TLM or RT stratified by their cancer’s T stage. 23 patients were treated with TLM, with 21 patients having a cancer staged as T1 and 2 as T2; and 52 patients were treated with RT, with 26 patients having a cancer staged as T1 and 26 as T2. A statistically significant difference in the distribution of T-stage across treatment group was observed (*χ* ^2^ = 11.3, *p* = 0.001)

**Fig. 3:**
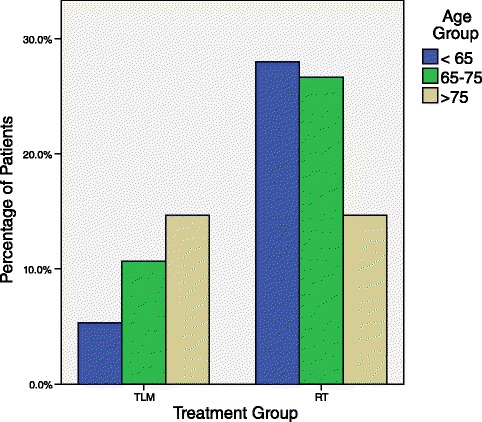
Distribution of patients by treatment group stratified by age group. This double bar graph represents the distribution of patients who underwent TLM or RT stratified by their age group. 23 patients were treated with TLM, with 4 less than 65 years old (5.3 %), 8 patients 65–75 years old (10.7 %), and 11 patients greater than 75 years old (14.7 %); and 52 patients were treated with RT, with 21 patients less than 65 years old (28 %), 20 patients 65–75 years old (26.7 %), and 11 greater than 75 years old (14.7 %). A statistically significant difference in the distribution of age group across treatment group was observed (*χ* ^2^ = 6.455, *p* = 0.04)

The extent of one’s cancer, often represented by T-stage can affect the survival of a patient, with more severe cancers often having a worse prognosis. When comparing T-stage to the treatment groups, TLM and RT, through a Chi-square analysis, a statistically significant relationship (*χ*^2^ = 11.3, *p* = 0.001) was observed, therefore making it necessary to stratify based on T stage (Fig. 2). Furthermore, in general, being older is associated with worse cancer survival rates, likely due to an overall better general health and response to treatments in younger patients [2]. Chi-square analysis of age-group versus treatment group revealed a statistically significant relationship (*χ*^2^ = 6.455, *p* = 0.04), thus making it reasonable to account for age as a confounder (Fig. 3). Additionally, gender could potentially alter the cancer specific survival rate, with worse results in males [3]. However, no relationship between treatment group and the distribution of gender was seen (*χ*^2^ = 0.034, *p* = 1.000).

Consequently, subsequent analyses for 5-year survival was stratified first by T-Stage. However only 2 patients whose cancer was categorized as T2 were treated with TLM (Fig. 3). With such a small sample being T2 and treated with TLM, a non-meaningful comparison would result from survival analysis stratified by T stage, and even less useful results if the analysis was further stratified by age group. Thus survival analysis of all patients in the sample population was conducted stratified by age-group only and a subgroup analysis of T1 patients stratified by age was examined so both age and T stage were accounted for in the analysis.

### Disease-specific survival

**Fig. 4:**
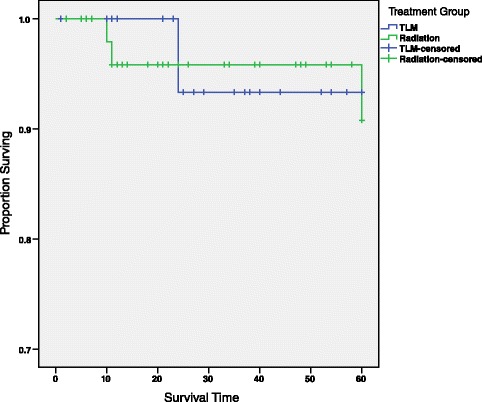
5-year disease-specific survival curve by treatment group. This survival curve depicts the 5-year disease-specific survival for all patients reported in this study, and is divided by treatment group, TLM and RT. No statistically significant relationship was observed between treatment group and 5-year survival (*χ* ^2^ < 0.001, *p* = 0.983). Censored patients are those whose did not die from their primary cancer but were lost to follow-up before the 5-year mark

Disease-specific 5-year survival rate is 93.3 % (σ = 0.064) and 90.8 % (σ = 0.056) for patients treated with TLM and RT, respectively (Fig. 4 and Table 3). The difference in survival between TLM and RT treatment groups was not statistically significant, *χ*^2^ < 0.001, *p* = 0.983. Additionally, we tested whether any variables not matched or controlled for skewed the results.

**Table 3:** 5-year disease-specific survival life-table

First-order Controls	Interval Start time	Number Entering Interval	Number Withdrawing during Interval	Number Exposed to Risk	Number of Terminal Events	Proportion Surviving	Cumulative Proportion Surviving at End of Interval	Std. Error of Cumulative Proportion Surviving at End of Interval
TLM	0	23	0	23.000	0	1.00	1.00	.00
	1	23	1	22.500	0	1.00	1.00	.00
	2	22	0	22.000	0	1.00	1.00	.00
	3	22	0	22.000	0	1.00	1.00	.00
	4	22	0	22.000	0	1.00	1.00	.00
	5	22	1	21.500	0	1.00	1.00	.00
	6	21	0	21.000	0	1.00	1.00	.00
	7	21	1	20.500	0	1.00	1.00	.00
	8	20	0	20.000	0	1.00	1.00	.00
	9	20	0	20.000	0	1.00	1.00	.00
	10	20	1	19.500	0	1.00	1.00	.00
	11	19	1	18.500	0	1.00	1.00	.00
	12	18	1	17.500	0	1.00	1.00	.00
	13	17	0	17.000	0	1.00	1.00	.00
	14	17	0	17.000	0	1.00	1.00	.00
	15	17	0	17.000	0	1.00	1.00	.00
	16	17	0	17.000	0	1.00	1.00	.00
	17	17	0	17.000	0	1.00	1.00	.00
	18	17	0	17.000	0	1.00	1.00	.00
	19	17	0	17.000	0	1.00	1.00	.00
	20	17	0	17.000	0	1.00	1.00	.00
	21	17	1	16.500	0	1.00	1.00	.00
	22	16	0	16.000	0	1.00	1.00	.00
	23	16	1	15.500	0	1.00	1.00	.00
	24	15	0	15.000	1	.93	.93	.06
	25	14	1	13.500	0	1.00	.93	.06
	26	13	0	13.000	0	1.00	.93	.06
	27	13	1	12.500	0	1.00	.93	.06
	28	12	0	12.000	0	1.00	.93	.06
	29	12	1	11.500	0	1.00	.93	.06
	30	11	0	11.000	0	1.00	.93	.06
	31	11	0	11.000	0	1.00	.93	.06
	32	11	0	11.000	0	1.00	.93	.06
	33	11	0	11.000	0	1.00	.93	.06
	34	11	0	11.000	0	1.00	.93	.06
	35	11	1	10.500	0	1.00	.93	.06
	36	10	0	10.000	0	1.00	.93	.06
	37	10	1	9.500	0	1.00	.93	.06
	38	9	1	8.500	0	1.00	.93	.06
	39	8	0	8.000	0	1.00	.93	.06
	40	8	1	7.500	0	1.00	.93	.06
	41	7	0	7.000	0	1.00	.93	.06
	42	7	0	7.000	0	1.00	.93	.06
	43	7	0	7.000	0	1.00	.93	.06
	44	7	1	6.500	0	1.00	.93	.06
	45	6	0	6.000	0	1.00	.93	.06
	46	6	0	6.000	0	1.00	.93	.06
	47	6	0	6.000	0	1.00	.93	.06
	48	6	0	6.000	0	1.00	.93	.06
	49	6	0	6.000	0	1.00	.93	.06
	50	6	0	6.000	0	1.00	.93	.06
	51	6	0	6.000	0	1.00	.93	.06
	52	6	1	5.500	0	1.00	.93	.06
	53	5	0	5.000	0	1.00	.93	.06
	54	5	1	4.500	0	1.00	.93	.06
	55	4	0	4.000	0	1.00	.93	.06
	56	4	0	4.000	0	1.00	.93	.06
	57	4	1	3.500	0	1.00	.93	.06
	58	3	0	3.000	0	1.00	.93	.06
	59	3	0	3.000	0	1.00	.93	.06
	60	3	3	1.500	0	1.00	.93	.06
Radiation	0	52	0	52.000	0	1.00	1.00	.00
	1	52	0	52.000	0	1.00	1.00	.00
	2	52	1	51.500	0	1.00	1.00	.00
	3	51	0	51.000	0	1.00	1.00	.00
	4	51	0	51.000	0	1.00	1.00	.00
	5	51	1	50.500	0	1.00	1.00	.00
	6	50	1	49.500	0	1.00	1.00	.00
	7	49	1	48.500	0	1.00	1.00	.00
	8	48	0	48.000	0	1.00	1.00	.00
	9	48	0	48.000	0	1.00	1.00	.00
	10	48	0	48.000	1	.98	.98	.02
	11	47	1	46.500	1	.98	.96	.03
	12	45	2	44.000	0	1.00	.96	.03
	13	43	1	42.500	0	1.00	.96	.03
	14	42	1	41.500	0	1.00	.96	.03
	15	41	0	41.000	0	1.00	.96	.03
	16	41	0	41.000	0	1.00	.96	.03
	17	41	0	41.000	0	1.00	.96	.03
	18	41	3	39.500	0	1.00	.96	.03
	19	38	0	38.000	0	1.00	.96	.03
	20	38	1	37.500	0	1.00	.96	.03
	21	37	1	36.500	0	1.00	.96	.03
	22	36	1	35.500	0	1.00	.96	.03
	23	35	0	35.000	0	1.00	.96	.03
	24	35	1	34.500	0	1.00	.96	.03
	25	34	0	34.000	0	1.00	.96	.03
	26	34	4	32.000	0	1.00	.96	.03
	27	30	0	30.000	0	1.00	.96	.03
	28	30	0	30.000	0	1.00	.96	.03
	29	30	0	30.000	0	1.00	.96	.03
	30	30	0	30.000	0	1.00	.96	.03
	31	30	0	30.000	0	1.00	.96	.03
	32	30	0	30.000	0	1.00	.96	.03
	33	30	1	29.500	0	1.00	.96	.03
	34	29	1	28.500	0	1.00	.96	.03
	35	28	0	28.000	0	1.00	.96	.03
	36	28	0	28.000	0	1.00	.96	.03
	37	28	0	28.000	0	1.00	.96	.03
	38	28	0	28.000	0	1.00	.96	.03
	39	28	2	27.000	0	1.00	.96	.03
	40	26	1	25.500	0	1.00	.96	.03
	41	25	0	25.000	0	1.00	.96	.03
	42	25	0	25.000	0	1.00	.96	.03
	43	25	0	25.000	0	1.00	.96	.03
	44	25	0	25.000	0	1.00	.96	.03
	45	25	0	25.000	0	1.00	.96	.03
	46	25	0	25.000	0	1.00	.96	.03
	47	25	1	24.500	0	1.00	.96	.03
	48	24	1	23.500	0	1.00	.96	.03
	49	23	1	22.500	0	1.00	.96	.03
	50	22	0	22.000	0	1.00	.96	.03
	51	22	0	22.000	0	1.00	.96	.03
	52	22	0	22.000	0	1.00	.96	.03
	53	22	1	21.500	0	1.00	.96	.03
	54	21	1	20.500	0	1.00	.96	.03
	55	20	0	20.000	0	1.00	.96	.03
	56	20	0	20.000	0	1.00	.96	.03
	57	20	0	20.000	0	1.00	.96	.03
	58	20	1	19.500	0	1.00	.96	.03
	59	19	0	19.000	0	1.00	.96	.03
	60	19	18	10.000	1	.90	.86	.09

This life-table shows the proportion of patients who experienced an event each time-interval (1 month). Additionally, the table indicates the amount of patients who were censored each month by reporting how many patients entered and left each interval

However, the log-rank statistic, adjusted for age group, revealed no significant relationship between treatment group and cancer related 5-year survival rate (*χ*^2^ = 0.347, *p* = 0.556). Additionally, the 5-year survival curve of T1 patients stratified by age group yielded no statistically significant relationship between treatment group and cancer related 5-year survival rate (*χ*^2^ = 0.033, *p* = 0.856).

### Disease-free survival

**Fig. 5:**
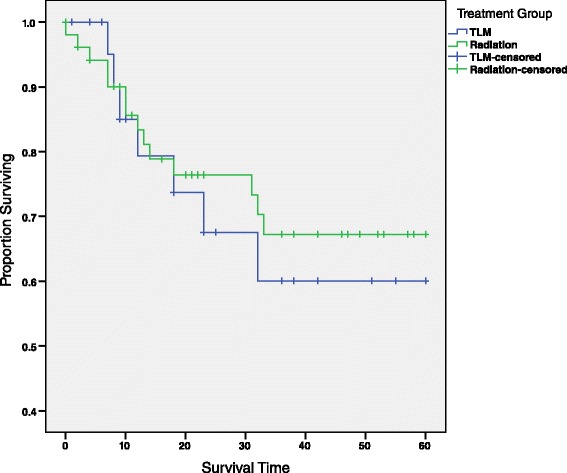
5-year disease-free survival curve by treatment group. This survival curve depicts the 5-year disease-free survival for all patients reported in this study, and is divided by treatment group, TLM and RT. No statistically significant relationship was observed between treatment group and 5-year survival (*χ* ^2^ = 0.19, *p* = 0.663). Censored patients are those whose cancer did not recur nor did they die from their primary cancer but were lost to follow-up before the 5-year mark

The disease free survival rate is 60.0 % (σ =0.121) for patients treated with TLM, and 67.2 % (σ = 0.074) for those who received RT (Fig. 5 and Table 4). This difference in survival was not statistically significant (*χ*^2^ = 0.19, *p* = 0.663). The same conclusion can be drawn from the log-rank statistic for the survival rate stratified by age-group (*χ*^2^ = 0.049, *p* = 0.824). Additionally, the 5-year disease free survival curve of T1 patients stratified by age group revealed no statistically significant difference between TLM and RT groups (*χ*^2^ = 1.034, *p* = 0.596).

**Table 4:** 5-year disease-free survival life-table

First-order Controls	Interval Start time	Number Entering Interval	Number Withdrawing during Interval	Number Exposed to Risk	Number of Terminal Events	Proportion Surviving	Cumulative Proportion Surviving at End of Interval	Std. Error of Cumulative Proportion Surviving at End of Interval
TLM	0	23	0	23.000	0	1.00	1.00	.00
	1	23	1	22.500	0	1.00	1.00	.00
	2	22	0	22.000	0	1.00	1.00	.00
	3	22	0	22.000	0	1.00	1.00	.00
	4	22	1	21.500	0	1.00	1.00	.00
	5	21	0	21.000	0	1.00	1.00	.00
	6	21	1	20.500	0	1.00	1.00	.00
	7	20	0	20.000	1	.95	.95	.05
	8	19	0	19.000	1	.95	.90	.07
	9	18	1	17.500	1	.94	.85	.08
	10	16	1	15.500	0	1.00	.85	.08
	11	15	0	15.000	0	1.00	.85	.08
	12	15	0	15.000	1	.93	.79	.09
	13	14	0	14.000	0	1.00	.79	.09
	14	14	0	14.000	0	1.00	.79	.09
	15	14	0	14.000	0	1.00	.79	.09
	16	14	0	14.000	0	1.00	.79	.09
	17	14	0	14.000	0	1.00	.79	.09
	18	14	1	13.500	1	.93	.73	.10
	19	12	0	12.000	0	1.00	.73	.10
	20	12	0	12.000	0	1.00	.73	.10
	21	12	0	12.000	0	1.00	.73	.10
	22	12	0	12.000	0	1.00	.73	.10
	23	12	1	11.500	1	.91	.67	.11
	24	10	0	10.000	0	1.00	.67	.11
	25	10	1	9.500	0	1.00	.67	.11
	26	9	0	9.000	0	1.00	.67	.11
	27	9	0	9.000	0	1.00	.67	.11
	28	9	0	9.000	0	1.00	.67	.11
	29	9	0	9.000	0	1.00	.67	.11
	30	9	0	9.000	0	1.00	.67	.11
	31	9	0	9.000	0	1.00	.67	.11
	32	9	0	9.000	1	.89	.60	.12
	33	8	0	8.000	0	1.00	.60	.12
	34	8	0	8.000	0	1.00	.60	.12
	35	8	0	8.000	0	1.00	.60	.12
	36	8	1	7.500	0	1.00	.60	.12
	37	7	0	7.000	0	1.00	.60	.12
	38	7	3	5.500	0	1.00	.60	.12
	39	4	0	4.000	0	1.00	.60	.12
	40	4	0	4.000	0	1.00	.60	.12
	41	4	0	4.000	0	1.00	.60	.12
	42	4	1	3.500	0	1.00	.60	.12
	43	3	0	3.000	0	1.00	.60	.12
	44	3	0	3.000	0	1.00	.60	.12
	45	3	0	3.000	0	1.00	.60	.12
	46	3	0	3.000	0	1.00	.60	.12
	47	3	0	3.000	0	1.00	.60	.12
	48	3	0	3.000	0	1.00	.60	.12
	49	3	0	3.000	0	1.00	.60	.12
	50	3	0	3.000	0	1.00	.60	.12
	51	3	1	2.500	0	1.00	.60	.12
	52	2	0	2.000	0	1.00	.60	.12
	53	2	0	2.000	0	1.00	.60	.12
	54	2	0	2.000	0	1.00	.60	.12
	55	2	1	1.500	0	1.00	.60	.12
	56	1	0	1.000	0	1.00	.60	.12
	57	1	0	1.000	0	1.00	.60	.12
	58	1	0	1.000	0	1.00	.60	.12
	59	1	0	1.000	0	1.00	.60	.12
	60	1	1	.500	0	1.00	.60	.12
Radiation	0	52	1	51.500	1	.98	.98	.02
	1	50	0	50.000	0	1.00	.98	.02
	2	50	1	49.500	1	.98	.96	.03
	3	48	0	48.000	0	1.00	.96	.03
	4	48	1	47.500	1	.98	.94	.03
	5	46	0	46.000	0	1.00	.94	.03
	6	46	0	46.000	0	1.00	.94	.03
	7	46	0	46.000	2	.96	.90	.04
	8	44	1	43.500	0	1.00	.90	.04
	9	43	2	42.000	0	1.00	.90	.04
	10	41	0	41.000	2	.95	.86	.05
	11	39	1	38.500	0	1.00	.86	.05
	12	38	0	38.000	1	.97	.83	.05
	13	37	0	37.000	1	.97	.81	.06
	14	36	0	36.000	1	.97	.79	.06
	15	35	0	35.000	0	1.00	.79	.06
	16	35	3	33.500	0	1.00	.79	.06
	17	32	0	32.000	0	1.00	.79	.06
	18	32	0	32.000	1	.97	.76	.06
	19	31	0	31.000	0	1.00	.76	.06
	20	31	1	30.500	0	1.00	.76	.06
	21	30	1	29.500	0	1.00	.76	.06
	22	29	2	28.000	0	1.00	.76	.06
	23	27	2	26.000	0	1.00	.76	.06
	24	25	0	25.000	0	1.00	.76	.06
	25	25	0	25.000	0	1.00	.76	.06
	26	25	0	25.000	0	1.00	.76	.06
	27	25	0	25.000	0	1.00	.76	.06
	28	25	0	25.000	0	1.00	.76	.06
	29	25	0	25.000	0	1.00	.76	.06
	30	25	0	25.000	0	1.00	.76	.06
	31	25	0	25.000	1	.96	.73	.07
	32	24	0	24.000	1	.96	.70	.07
	33	23	0	23.000	1	.96	.67	.07
	34	22	0	22.000	0	1.00	.67	.07
	35	22	0	22.000	0	1.00	.67	.07
	36	22	1	21.500	0	1.00	.67	.07
	37	21	0	21.000	0	1.00	.67	.07
	38	21	1	20.500	0	1.00	.67	.07
	39	20	0	20.000	0	1.00	.67	.07
	40	20	0	20.000	0	1.00	.67	.07
	41	20	0	20.000	0	1.00	.67	.07
	42	20	1	19.500	0	1.00	.67	.07
	43	19	0	19.000	0	1.00	.67	.07
	44	19	0	19.000	0	1.00	.67	.07
	45	19	0	19.000	0	1.00	.67	.07
	46	19	2	18.000	0	1.00	.67	.07
	47	17	1	16.500	0	1.00	.67	.07
	48	16	0	16.000	0	1.00	.67	.07
	49	16	1	15.500	0	1.00	.67	.07
	50	15	0	15.000	0	1.00	.67	.07
	51	15	0	15.000	0	1.00	.67	.07
	52	15	2	14.000	0	1.00	.67	.07
	53	13	1	12.500	0	1.00	.67	.07
	54	12	0	12.000	0	1.00	.67	.07
	55	12	0	12.000	0	1.00	.67	.07
	56	12	0	12.000	0	1.00	.67	.07
	57	12	1	11.500	0	1.00	.67	.07
	58	11	1	10.500	0	1.00	.67	.07
	59	10	0	10.000	0	1.00	.67	.07
	60	10	10	5.000	0	1.00	.67	.07

This life-table shows the proportion of patients who experienced an event each time-interval (1 month). Additionally, the table indicates the amount of patients who were censored each month by reporting how many patients entered and left each interval

### Total laryngectomy-free survival

**Fig. 6:**
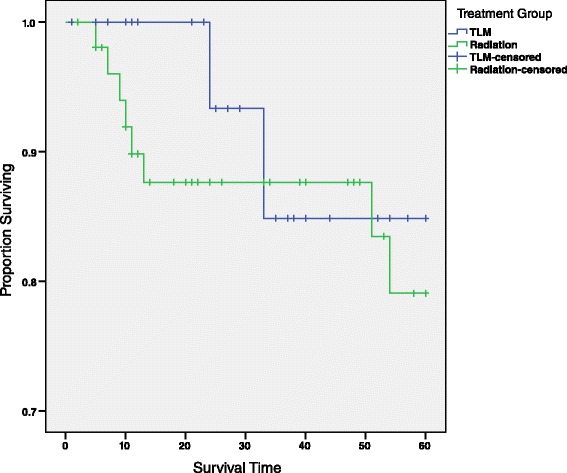
5-year total laryngectomy-free survival curve by treatment group. This survival curve depicts the 5-year total laryngectomy-free survival for all patients reported in this study, and is divided by treatment group, TLM and RT. No statistically significant relationship was observed between treatment group and 5-year survival (*χ* ^2^ = 0.289, *p* = 0.591). Censored patients are those whose did not receive a total laryngectomy, nor did they die from their primary cancer, but were lost to follow-up before the 5-year mark

The total laryngectomy-free survival rate is 84.1 % (σ = 0.1) and 79.1 % (σ = 0.072) for patients’ early glottic cancer treated by TLM and RT, respectively (Fig. 6 and Table 5). This variation in the survival rates was not statistically significant (*χ*^2^ = 0.289, *p* = 0.591). Furthermore, upon stratifying by age, log-rank statistics reveal no significant difference in the survival rates (*χ*^2^ = 0.235, *p* = 0.628). This conclusion is congruent to the results of the laryngectomy free survival analysis of T1 patients stratified by age-group (*χ*^2^ = 1.692, *p* = 0.429).

**Table 5:** 5-year total laryngectomy-free survival life-table

First-order Controls	Interval Start time	Number Entering Interval	Number Withdrawing during Interval	Number Exposed to Risk	Number of Terminal Events	Proportion Surviving	Cumulative Proportion Surviving at End of Interval	Std. Error of Cumulative Proportion Surviving at End of Interval
TLM	0	23	0	23.000	0	1.00	1.00	.00
	1	23	1	22.500	0	1.00	1.00	.00
	2	22	0	22.000	0	1.00	1.00	.00
	3	22	0	22.000	0	1.00	1.00	.00
	4	22	0	22.000	0	1.00	1.00	.00
	5	22	1	21.500	0	1.00	1.00	.00
	6	21	0	21.000	0	1.00	1.00	.00
	7	21	1	20.500	0	1.00	1.00	.00
	8	20	0	20.000	0	1.00	1.00	.00
	9	20	0	20.000	0	1.00	1.00	.00
	10	20	1	19.500	0	1.00	1.00	.00
	11	19	1	18.500	0	1.00	1.00	.00
	12	18	1	17.500	0	1.00	1.00	.00
	13	17	0	17.000	0	1.00	1.00	.00
	14	17	0	17.000	0	1.00	1.00	.00
	15	17	0	17.000	0	1.00	1.00	.00
	16	17	0	17.000	0	1.00	1.00	.00
	17	17	0	17.000	0	1.00	1.00	.00
	18	17	0	17.000	0	1.00	1.00	.00
	19	17	0	17.000	0	1.00	1.00	.00
	20	17	0	17.000	0	1.00	1.00	.00
	21	17	1	16.500	0	1.00	1.00	.00
	22	16	0	16.000	0	1.00	1.00	.00
	23	16	1	15.500	0	1.00	1.00	.00
	24	15	0	15.000	1	.93	.93	.06
	25	14	1	13.500	0	1.00	.93	.06
	26	13	0	13.000	0	1.00	.93	.06
	27	13	1	12.500	0	1.00	.93	.06
	28	12	0	12.000	0	1.00	.93	.06
	29	12	1	11.500	0	1.00	.93	.06
	30	11	0	11.000	0	1.00	.93	.06
	31	11	0	11.000	0	1.00	.93	.06
	32	11	0	11.000	0	1.00	.93	.06
	33	11	0	11.000	1	.91	.85	.10
	34	10	0	10.000	0	1.00	.85	.10
	35	10	1	9.500	0	1.00	.85	.10
	36	9	0	9.000	0	1.00	.85	.10
	37	9	1	8.500	0	1.00	.85	.10
	38	8	1	7.500	0	1.00	.85	.10
	39	7	0	7.000	0	1.00	.85	.10
	40	7	1	6.500	0	1.00	.85	.10
	41	6	0	6.000	0	1.00	.85	.10
	42	6	0	6.000	0	1.00	.85	.10
	43	6	0	6.000	0	1.00	.85	.10
	44	6	1	5.500	0	1.00	.85	.10
	45	5	0	5.000	0	1.00	.85	.10
	46	5	0	5.000	0	1.00	.85	.10
	47	5	0	5.000	0	1.00	.85	.10
	48	5	0	5.000	0	1.00	.85	.10
	49	5	0	5.000	0	1.00	.85	.10
	50	5	0	5.000	0	1.00	.85	.10
	51	5	0	5.000	0	1.00	.85	.10
	52	5	1	4.500	0	1.00	.85	.10
	53	4	0	4.000	0	1.00	.85	.10
	54	4	1	3.500	0	1.00	.85	.10
	55	3	0	3.000	0	1.00	.85	.10
	56	3	0	3.000	0	1.00	.85	.10
	57	3	1	2.500	0	1.00	.85	.10
	58	2	0	2.000	0	1.00	.85	.10
	59	2	0	2.000	0	1.00	.85	.10
	60	2	2	1.000	0	1.00	.85	.10
Radiation	0	52	0	52.000	0	1.00	1.00	.00
	1	52	0	52.000	0	1.00	1.00	.00
	2	52	1	51.500	0	1.00	1.00	.00
	3	51	0	51.000	0	1.00	1.00	.00
	4	51	0	51.000	0	1.00	1.00	.00
	5	51	1	50.500	1	.98	.98	.02
	6	49	1	48.500	0	1.00	.98	.02
	7	48	0	48.000	1	.98	.96	.03
	8	47	0	47.000	0	1.00	.96	.03
	9	47	0	47.000	1	.98	.94	.03
	10	46	1	45.500	1	.98	.92	.04
	11	44	1	43.500	1	.98	.90	.04
	12	42	1	41.500	0	1.00	.90	.04
	13	41	0	41.000	1	.98	.88	.05
	14	40	1	39.500	0	1.00	.88	.05
	15	39	0	39.000	0	1.00	.88	.05
	16	39	0	39.000	0	1.00	.88	.05
	17	39	0	39.000	0	1.00	.88	.05
	18	39	3	37.500	0	1.00	.88	.05
	19	36	0	36.000	0	1.00	.88	.05
	20	36	1	35.500	0	1.00	.88	.05
	21	35	1	34.500	0	1.00	.88	.05
	22	34	1	33.500	0	1.00	.88	.05
	23	33	0	33.000	0	1.00	.88	.05
	24	33	1	32.500	0	1.00	.88	.05
	25	32	0	32.000	0	1.00	.88	.05
	26	32	3	30.500	0	1.00	.88	.05
	27	29	0	29.000	0	1.00	.88	.05
	28	29	0	29.000	0	1.00	.88	.05
	29	29	0	29.000	0	1.00	.88	.05
	30	29	0	29.000	0	1.00	.88	.05
	31	29	0	29.000	0	1.00	.88	.05
	32	29	0	29.000	0	1.00	.88	.05
	33	29	1	28.500	0	1.00	.88	.05
	34	28	1	27.500	0	1.00	.88	.05
	35	27	0	27.000	0	1.00	.88	.05
	36	27	0	27.000	0	1.00	.88	.05
	37	27	0	27.000	0	1.00	.88	.05
	38	27	0	27.000	0	1.00	.88	.05
	39	27	2	26.000	0	1.00	.88	.05
	40	25	1	24.500	0	1.00	.88	.05
	41	24	0	24.000	0	1.00	.88	.05
	42	24	0	24.000	0	1.00	.88	.05
	43	24	0	24.000	0	1.00	.88	.05
	44	24	0	24.000	0	1.00	.88	.05
	45	24	0	24.000	0	1.00	.88	.05
	46	24	0	24.000	0	1.00	.88	.05
	47	24	1	23.500	0	1.00	.88	.05
	48	23	1	22.500	0	1.00	.88	.05
	49	22	1	21.500	0	1.00	.88	.05
	50	21	0	21.000	0	1.00	.88	.05
	51	21	0	21.000	1	.95	.83	.06
	52	20	0	20.000	0	1.00	.83	.06
	53	20	1	19.500	0	1.00	.83	.06
	54	19	0	19.000	1	.95	.79	.07
	55	18	0	18.000	0	1.00	.79	.07
	56	18	0	18.000	0	1.00	.79	.07
	57	18	0	18.000	0	1.00	.79	.07
	58	18	1	17.500	0	1.00	.79	.07
	59	17	0	17.000	0	1.00	.79	.07
	60	17	17	8.500	0	1.00	.79	.07

This life-table shows the proportion of patients who experienced an event each time-interval (1 month). Additionally, the table indicates the amount of patients who were censored each month by reporting how many patients entered and left each interval

## Discussion

An analysis of early glottic cancer patients at Sunnybrook did not demonstrate a difference in the 5-year disease-specific survival, disease-free survival, and total laryngectomy-free survival, between the RT and TLM groups. Though these results clash with those reported by Hartl et al (2012) and van Gogh et al (2012), they are consistent with a systematic review by Loon et al (2012) and meta-analysis by Higgins (2011), and retrospective cohort study by Remmelts et al (2012) [4–7]. The literature reports 5-year survival rates ranging from 75 to 93 % [4, 7–16], which is consistent with the results reported in this paper [4, 7–16].

The consideration of confounding variables was imperative to this study, especially as very few studies consider the potential effects various T stages, age and gender can have on the data. Thus to avoid skewed results, variables were tested to see if they had some relationship with the treatment groups to allow for stratification and more accurate results. Though no significant relationship was found between treatment modality and survival even after stratification, accounting for confounding variables helped to ensure the verity of the results.

The results of this study add to the existing literature that suggests TLM and RT are both effective treatment modalities for early larynx cancer, and provide similar results in terms of preventing cancer related deaths. This result is important as it can help in the decision process of making recommendations for patients’ treatment plans. One factor in choosing which treatments should be used is the cost. Since the literature tends to agree that neither treatment produces better oncologic [6–8, 17] or functional outcomes [7, 9, 10, 18, 19] making decision based on cost is reasonable.

A meta-analysis by Higgins (2011) shows that in Ontario, TLM is more cost-effective when compared to RT [7]. It is suggested that is largely due to TLM having more affordable options for salvage treatment in comparison to RT [7]. These results were also supported by a Chinese meta-analysis suggesting that TLM is significantly cheaper than RT in the context of early glottis cancer. Furthermore, they conclude that TLM should be used over RT due the drastic difference in prices [9]. Merrot et al. (2010) also indicate similar conclusions based on a meta-analysis of French healthcare facilities [10]. These studies provide a good indication of which treatment is more cost-effective, and the fact that three meta-analyses from different regions suggest similar results, is encouraging. However, it could be dangerous to apply the conclusions of these studies to different jurisdictions. Thus, it is encouraged for other healthcare networks to conduct similar reviews to help decision makers in their respective dominions.

Limitations of this study are intrinsically linked to its retrospective nature. Radiation dosage were not standardized or controlled for. Furthermore, patients were not randomized into their treatment group. Thus the effectiveness of each treatment may be skewed as a patient’s treatment was selected since it was thought by the patient and the clinical team that it would yield better results for that patient. As well patients’ comorbidities could have affected their survival and treatment selection. However, ethically, it may be difficult to conduct a perfect randomized study, since a certain treatment for a specific patient may go against the clinician’s and patient’s beliefs about what the best treatment is. Moreover, there was an imbalance of patients in each treatment group. This variance could have affected the observed results. However, preventive measures were in place to minimize such an effect by stratifying the results and conducting subgroup analysis. Additionally, the results only reflect a single institution experience, thus making the results dependent on the clinician’s individual expertise. Thus, multi-institutional studies or meta-analyses are necessary to make more established conclusions.

## Conclusion

No difference was demonstrated in the 5-year disease-specific survival, disease-free survival, and total laryngectomy-free survival, between the RT and TLM treatment groups. Additionally, both groups showed similar 5-year survival before and after stratifying by confounding variables; age and T stage. The significance of this result, when combined with the existing evidence in the literature, is that decisions on which treatment should be prescribed may not need to be centered around efficacy, but rather on other meaningful factors such as voice quality, patient values, or cost. Despite being limited by the retrospective study design, this study is the largest Canadian comparison of consecutive patients treated with either RT or TLM, and stands out for considering potential confounders as means for ensuring the validity of the results.

## Abbreviations

RT, radiation therapy; TLM, trans-oral laser microsurgery.
